# Facioscapulohumeral dystrophy: the path to consensus on pathophysiology

**DOI:** 10.1186/2044-5040-4-12

**Published:** 2014-06-10

**Authors:** Rabi Tawil, Silvère M van der Maarel, Stephen J Tapscott

**Affiliations:** 1 Department of Neurology, University of Rochester, Rochester, NY 14642, USA; 2 Department of Human Genetics, Leiden University Medical Center, 2333 ZA, Leiden, The Netherlands; 3 Divisions of Human Biology and Clinical Research, Fred Hutchinson Cancer Research Center, Seattle, WA 98109, USA; 4 Department of Neurology, University of Washington, Seattle, WA 98105, USA; 5 Fred Hutchinson Cancer Research Center, 1100 Fairview Avenue North, Seattle, WA 98109, USA

**Keywords:** Facioscapulohumeral muscular dystrophy, DUX4, SMCHD1, Epigenetic, Tandem repeat sequences

## Abstract

Although the pathophysiology of facioscapulohumeral dystrophy (FSHD) has been controversial over the last decades, progress in recent years has led to a model that incorporates these decades of findings and is gaining general acceptance in the FSHD research community. Here we review how the contributions from many labs over many years led to an understanding of a fundamentally new mechanism of human disease. FSHD is caused by inefficient repeat-mediated epigenetic repression of the D4Z4 macrosatellite repeat array on chromosome 4, resulting in the variegated expression of the *DUX4* retrogene, encoding a double-homeobox transcription factor, in skeletal muscle. Normally expressed in the testis and epigenetically repressed in somatic tissues, DUX4 expression in skeletal muscle induces expression of many germline, stem cell, and other genes that might account for the pathophysiology of FSHD. Although some disagreements regarding the details of mechanisms remain in the field, the coalescing agreement on a central model of pathophysiology represents a pivot-point in FSHD research, transitioning the field from discovery-oriented studies to translational studies aimed at developing therapies based on a sound model of disease pathophysiology.

## Introduction

The mutation causing the most common form of facioscapulohumeral muscular dystrophy (FSHD) was identified over 20 years ago, yet for many of the ensuing years there was little or no consensus in the scientific community regarding the molecular pathophysiology of the disease. Unlike other disease-causing mutations that disrupt the normal function of an identified gene, FSHD is caused by the loss of a subset of repeat units in the D4Z4 macrosatellite repeat array on chromosome 4 that does not disrupt the structure of any gene. This led to many different hypotheses regarding the mechanism of FSHD pathophysiology, and many disagreements among the researchers in this field. The absence of a generally accepted model for FSHD pathophysiology made it difficult to get support for research in FSHD and limited the interest in therapeutic development. Over the last several years, several key advances in FSHD research, many made possible by the steady improvement in technology, have identified the molecular and genetic causes of FSHD and clarified the mechanisms of pathophysiology, leading to the discovery that FSHD is a disease of inefficient repeat-mediated epigenetic repression of the *DUX4* retrogene embedded in the D4Z4 repeat units. Here we will review the line of experimental evidence that gradually led to this model of FSHD pathophysiology. As this model gains general acceptance in the field, greater attention and resources can now be devoted to therapeutic development, indicating that we have come to a pivotal moment in FSHD research.

## Review

### Clinical features of FSHD

One of the earliest descriptions of FSHD was published in 1885 by the French neurologists Landouzy and Dejerine [1]. The clinical syndrome was further characterized in a subsequent study of large Mormon families in Utah by Tyler and Stephens [2]. The most comprehensive study, describing the salient features of FSHD, was written by George Padberg in 1982 as a dissertation thesis (http://hdl.handle.net/1887/25818). In the vast majority of cases, FSHD is an autosomal dominant disease with a high frequency of *de novo* mutations [3]. However, about 5% of patients with clinical FSHD, termed FSHD2, are genetically distinct with a more complex digenic inheritance pattern (see section on FSHD2) [4]. To date, based on a relatively small study, FSHD1 and 2 appear to be clinically indistinguishable; however, larger studies are needed to confirm this observation [5].

Current estimates of the prevalence of FSHD range from 1:14,000 to 1:20,000 [6-9]. The age at disease onset ranges from infancy to middle age with the majority becoming symptomatic in the second and third decade of life. Early estimates of disease penetrance were 95% by the age of 20 years but recent studies suggest that the penetrance might be lower at this age [10,11]. In most instances, FSHD presents with a distinct, regional, often asymmetric muscle weakness starting rostrally in the face and shoulder muscles and progressing caudally over time to involve the trunk and leg muscles [3]. The early involvement of the periscapular muscles result in the distinctive profile of the shoulders of patients with FSHD with scapular winging, straight clavicles, and rounded shoulders [3]. The presence of a combination of scapular winging and facial weakness without other signs of muscle involvement, and an autosomal dominant family history, makes the diagnosis of FSHD all but certain. As the disease progresses, the muscles of the trunk and lower extremities become involved. Unlike other muscular dystrophies the extraocular muscles, pharyngeal muscles, and the cardiac muscle are spared [3]. Although rare, two distinguishing extramuscular manifestations in FSHD are the presence of a progressive high frequency hearing loss and a retinal exudative retinopathy (Coats disease), which if untreated can lead to blindness [12-15]. Symptomatic hearing loss and retinal vascular disease occurs almost exclusively in FSHD individuals with only one to three residual D4Z4 repeats (see nex section) and it is estimated that only about 1% of patients with FSHD develop Coats disease [13].

The spectrum of disease severity in FSHD varies widely with approximately 20% of genetically affected individuals remaining asymptomatic. Disease progression in general is relatively slow with estimates of an average loss of 5% of total strength per year as measured by manual muscle testing or quantitative myometry [13,16]. As a group, women are less severely affected and tend to have a later age at disease onset [13]. About 20% of patients with FSHD above the age of 50 years become wheelchair dependent and patients with the smallest residual repeat arrays are most at risk of wheelchair dependence [13,16]. In general life expectancy is not reduced in FSHD although about 1% of individuals can develop severe restrictive lung disease requiring the use of a ventilator [17].

In general, FSHD muscle does not show distinguishing, disease-specific characteristics on histopathologic examination. Unlike most dystrophies associated with structural protein defects, in FSHD the early myopathic changes are mild with relatively little fibrosis, muscle fiber hypertrophy, or central nucleation. Up to one-third of FSHD muscle biopsies show variable amounts of endomysial inflammation, often surrounding small endomysial blood vessels [18,19]. The inflammatory infiltrates are predominantly CD8+ with more prominent CD4+ T cells in the perivascular infiltrates [18,20]. Unlike polymyositis and inclusion body myositis, there does not appear to be a cytotoxic T-cell mediated muscle fiber injury as no invasion of non-necrotic fibers is observed in FSHD. Other dystrophies, such as Duchenne dystrophy and dysferlinopathies, are associated with inflammatory infiltrates but the predilection of the inflammatory infiltrates for the perivascular regions is unique to FSHD. This pathologic finding coupled with the occurrence of a retinal vasculopathy with an inflammatory component in FSHD and the demonstration of dysregulated vascular genes in FSHD muscle has raised the possibility that the T-cell mediated response may be directed against blood vessels [18,21].

### Loss of a subset of D4Z4 macorsatellite repeats causes FSHD

FSHD was one of the first Mendelian disorders mapped by the use of microsatellite markers to the distal end of the long arm of chromosome 4 in a cohort of Dutch FSHD families in 1990 [22]. This mapping to 4q35 was soon confirmed by others [23]. Further studies showed that in most families FSHD was genetically linked to a size reduction of a polymorphic *Eco*RI fragment below a threshold of 38 kb. Both in FSHD families and in sporadic cases, FSHD was consistently linked to this *Eco*RI fragment of variably reduced size [24,25]. Soon thereafter, the nature of this enigmatic observation was resolved by showing that the *Eco*RI fragment contained a tandem array of a variable number of 3.3 kb units, named D4Z4 units, in a head-to-tail orientation [26]. The DNA content of the D4Z4 unit is complex and contains sequences typically found in constitutive heterochromatin, but each unit also contains a copy of a homeobox sequence that was later characterized as the *DUX4* retrogene [27-30].

D4Z4-like sequences are distributed over the genome and can be found in pericentromeric and subtelomeric domains, but in the subtelomeres of chromosomes 4q and 10q the D4Z4 units are arranged in perfect tandem arrays [31,32]. Within the control population, the D4Z4 repeat array on chromosome 4 varies between 11 and 100 units, while the array on chromosome 10 can vary from one to 100 units [33]. Most patients with FSHD1 have one array of one to 10 units on chromosome 4 [34]; some patients with clinical features resembling FSHD have been reported carrying arrays of 11 units [35]. In approximately half of the *de novo* families, the mutation arose by a mitotic contraction of the D4Z4 repeat array leading to the presence of somatic mosaicism for the FSHD1 mutation in the proband, or in one of the clinically unaffected parents [36].

Units within an array are highly homologous, however, sequence polymorphisms between the units derived from chromosome 4 or 10 allow for correct chromosomal assignment. Generally, chromosome 10-derived units are uniquely sensitive to digestion by the endonuclease *Bln*I while chromosome 4-derived units are sensitive to digestion by *Xap*I [37,38]. This differential sensitivity to these endonucleases, and other polymorphisms that were discovered later in and around the D4Z4 repeat, facilitated studies into the dynamic nature of these repeats on both chromosomes. Based on several population studies a model emerged in which at least four subtelomeric exchanges between chromosomes 4 and 10 occurred during recent hominoid evolution leading to a complex picture ranging from individuals having the expected genetic configuration of chromosome 4-type repeats on chromosome 4 and 10-type repeats on chromosome 10, to individuals having translocated repeat arrays on either chromosome, to individuals showing evidence for carrying repeat arrays that are mixtures of 4-type and 10-type repeat units [33,39-41].

Despite the apparently dynamic behavior of these subtelomeric repeat arrays, D4Z4 repeat contractions on chromosome 4, but not on chromosome 10, were consistently identified in FSHD patients. This suggested that unique genetic features on chromosome 4 were necessary to cause FSHD, or alternatively, that sequences on chromosome 10 were protecting from pathogenicity. Moreover, at least one D4Z4 unit was necessary to cause FSHD since monosomy of the distal end of chromosome 4, including the D4Z4 repeat array, did not result in FSHD [42]. Because of the high homology of the repeats on chromosomes 4 and 10, and the 40 kb sequences proximal to the repeat, different models were proposed to explain the apparent chromosome 4 specificity. One model predicted that the D4Z4 repeat array acts as a barrier between the heterochromatic telomere, at 40 to 60 kb distance of the repeat, and proximal sequences. Upon repeat contraction, it was proposed that this barrier function becomes incomplete leading the spreading of heterochromatin in the centromeric direction and subsequent silencing of proximally located genes that are unique to chromosome 4. A second model predicted that the supposedly heterochromatic nature of D4Z4 itself was responsible for the regulation of cis-located genes by a looping or spreading mechanism and that upon contraction this regulation was impaired leading to the ectopic expression of proximally located genes on chromosome 4.

These models initiated the search for closely mapped genes and resulted in the identification of three genes and one pseudogene within an interval of 120 kb from the repeat. These included FSHD candidate region gene 1 (*FRG1*) at 120 kb distance from the repeat, the *TUBB4q* pseudogene at 80 kb distance, the *DUX4C* gene originating from an inverted and incomplete copy of the D4Z4 unit located at the boundary of the homologous regions on chromosomes 4 and 10, and *FRG2* at 35 kb distance and also present on chromosome 10 [43-46].

Many studies addressed the deregulation of proximally located genes with inconclusive results. An initial study reported the repeat length and distance dependent upregulation of *FRG1* and *FRG2* in muscle of FSHD patients [47]. However, follow-up studies by different groups and different techniques failed to confirm the upregulation of *FRG1* in FSHD muscle [21,48-50]. Also, chromatin studies of the D4Z4 repeat and immediately proximal sequences did not find evidence for a spreading of heterochromatin mechanism from the D4Z4 repeat, although some evidence for a role of D4Z4 in controlling *FRG1* expression by a looping mechanism was reported [48,51,52].

Although *FRG2* was consistently reported to be overexpressed from chromosomes 4 and 10 in FSHD muscle [41,44,48], its involvement in FSHD was challenged by the identification of an FSHD1 family in which the deletion not only involved the D4Z4 repeat array, but also eliminated *FRG2* and *DUX4C* from the disease allele [44,47,50,53]. This suggested that, although the upregulation of *FRG2* in FSHD remains poorly explained, it does not likely play an important role in FSHD pathology.

An observation that challenged both models was the discovery of two major chromosome 4 haplotypes, called 4A and 4B [54]. These two major variants of the distal end of chromosome 4 vary by small polymorphisms proximal and distal to the repeat, as well as in the repeat array itself. An important difference between the 4A and 4B variants is that the 4A variant contains a distal sequence called pLAM immediately followed by a beta satellite repeat, which are both absent on 4B chromosomes. Using probes that recognize sequences distal to the repeat, it was shown that both variants are almost equally prevalent in the population, but that only D4Z4 repeat contractions on 4A chromosomes are associated with FSHD, while contractions on 4B chromosomes do not cause disease [55,56]. This observation was difficult to unify with either of the two prevailing hypotheses explaining the chromosome 4 specificity.

### Coding and non-coding RNAs are produced from the D4Z4 region

**Figure 1:**
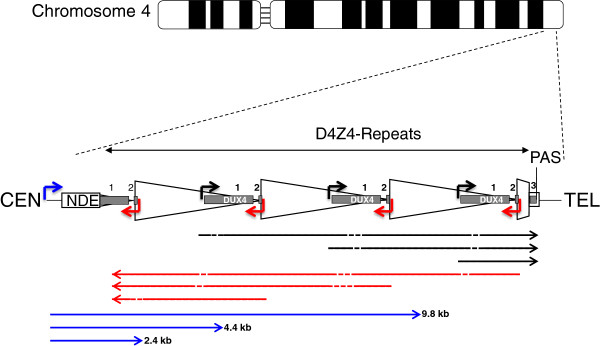
**Schematic of D4Z4 locus on chromosome 4.** The D4Z4 locus is in the sub-telomeric region of 4q. The figure shows a three repeat D4Z4 array. CEN indicates the centromeric end and TEL indicates the telomeric end. The *DUX4* gene is shown as a gray rectangle with exon 1 and exon 2 in each repeat and exon 3 in the pLAM region telomeric to the last partial repeat (numbered 1, 2, and 3). PAS indicates the polyadenylation site on the permissive 4qA allele that is not present on the non-permissive 4qB allele or on chromosome 10. The arrowed lines represent: Blue, DBE-T transcripts (2.4, 4.4, and 9.8 kb) found in FSHD cells and reported to de-repress DUX4 expression [60]; Black and red, transcripts in the sense and anti-sense direction were detected in both FSHD and control cells [29] and might originate from the mapped sense promoters (black) [29,30] and anti-sense promoters (red) [59] with dashed lines indicating areas that might be degraded or produce si-like small RNAs. NDE, non-deleted element identified as the transcription start site for the DBE-T transcripts [60].

As noted above, each of the D4Z4 units on chromosome 4 and 10 had been found to contain a copy of the *DUX4* retrogene. Because the DUX4 open reading frame (ORF) was conserved [27,57], there was debate regarding whether this was the result of positive selection for the function of the protein, particularly because DUX4 mRNA had not been detected in any primate tissues. The emerging model of RNA-mediated epigenetic repression of repetitive elements in model organisms also led to the hypothesis that D4Z4 RNA transcription might be associated with the repeat heterochromatin, however, RT-PCR and RNA polymerase II chromatin immunoprecipitation found little-or-no transcription of this region in cells from control or FSHD individuals [58]. Subsequently, Dixit *et al.*[30] used transfections of the genomic locus to identify DUX4 mRNA transcripts that were both spliced and poly-adenylated at a site distal to the last D4Z4 unit, in a region referred to as pLAM, and RT-PCR was used to identify this mRNA in FSHD muscle cells (Figure 1).

The production of a DUX4 mRNA with a poly-adenylation sequence in the pLAM region was confirmed in a study that also showed evidence for long transcripts through the D4Z4 repeats in both the sense and anti-sense directions [29]. Some of the sense transcripts from internal repeats were identified that had spliced into the pLAM exon containing the poly-adenylation sequence, indicating long transcripts spanning multiple D4Z4 units. In addition, small RNA fragments consistent with siRNAs or miRNAs were shown to be generated from these long transcripts, suggesting a possible role of RNA transcripts in epigenetic silencing of the locus as had been previously suggested, or possibly with other biological effects that can be mediated by small si or miRNAs.

The Snider *et al.* study [29] documented bidirectional RNA transcripts extending through multiple D4Z4 units and mapped the transcription start site of the *DUX4* containing transcript to a region immediately upstream of the DUX4 ORF. Block *et al.*[59] showed that a region of D4Z4 distal to the *DUX4* retrogene initiated transcripts in the anti-sense direction. Together these results suggest that transcripts might originate in each unit either near the promoter region of the DUX4 ORF or at a distal region and extend in each direction through multiple repeats. A more recent study focused on the long sense transcript through the D4Z4 units and called it a long non-coding RNA named DBE-T [60]. The sequence of the D4Z4 has many extended ORFs but the non-coding appellation might have been chosen because of the demonstration that the transcript was chromatin associated. The origin of this DBE-T transcript was mapped to the region centromeric to the D4Z4 repeats rather than to the promoters within D4Z4 described previously [29,30,59]. DBE-T was shown to be produced from FSHD and not control alleles, to recruit the ASH1L member of the trithorax group to the D4Z4 region, and to de-repress transcription of the DUX4 mRNA. Although the Cabianca et al. study [60] implicated a polycomb-mediated silencing of the non-contracted D4Z4 array, consistent with other prior studies, it differs from other models in that it identified a transcript originating in the region centromeric to the D4Z4 array that was selectively expressed in FSHD cells and necessary for de-repression of the locus, rather than the prior study identifying transcripts originating in the D4Z4 units in both control and FSHD cells that might contribute to RNA silencing of the locus [29] (see Figure 1). Further studies are necessary to determine which of these two distinct models is correct.

### Variegated expression of DUX4 in FSHD muscle

In contrast to some of the uncertainties regarding the molecular details of RNA-mediated regulation of D4Z4 transcription, the expression of a DUX4 mRNA as a cause of FSHD has gained further support. While the Dixit *et al.* study [30] identified a poly-adenylated DUX4 mRNA only in FSHD muscle, the Snider *et al.* study [29] identified DUX4 mRNA and other RNA transcripts in skeletal muscle cells from both control and FSHD affected individuals, albeit at higher levels in FSHD samples. In addition this study identified several different splice forms of the DUX4 mRNA, including one that would produce the full-length predicted ORF (DUX4-fl) and one that would produce a truncated protein that lacked the carboxy-terminal region (DUX4-s). A subsequent study [61] designed to measure the relative abundance of the DUX4-fl and DUX4-s mRNAs in skeletal muscle cells and muscle biopsies showed that the control biopsies expressed very low amounts of the DUX4-s transcript encoding the truncated protein, whereas the FSHD samples expressed very low amounts of the DUX4-fl and sometimes the DUX4-s as well, indicating that the DUX4-fl mRNA was correlated with FSHD and might be a cause for FSHD.

The transcripts for both DUX4-fl and DUX4-s, however, were at extremely low abundance, requiring high cycle PCR for reliable detection. It was unclear how such a low abundance mRNA, present at less than one copy per nucleus, could cause the disease. This was addressed using small-pool PCR and immunodetection of the DUX4 protein to show that a small subset of nuclei were producing relatively abundant DUX4-fl mRNA and protein with associated nuclear changes consistent with a pre-apoptotic effect [61]. Therefore, the low levels of DUX4-fl mRNA in FSHD muscle represented relatively high expression from a small subset of nuclei consistent with variegated expression patterns previously associated with repeat-mediated epigenetic repression [62]. In cultured mono-nuclear replicating myoblasts, isolated cells were found to express DUX4, indicating that the repression of DUX4 was occasionally lost and resulted in a burst of DUX4 expression with either subsequent silencing or the death of the cell. Myotubes have multiple nuclei in the same cell and expression of an mRNA from one nucleus will distribute the mRNA and protein to adjacent nuclei [63] and, indeed, the myotubes with a nucleus expressing DUX4 show the distribution of the protein to a regional domain of nuclei [64].

### Genetic confirmation that DUX4 mRNA causes FSHD

The previously mentioned observation that D4Z4 repeat contractions on 4A chromosomes were associated with FSHD, while contractions on 4B chromosomes did not cause disease [55,56] led to the genetic confirmation that the DUX4 mRNA was a necessary cause of FSHD [65]. First, a moderately polymorphic imperfect dinucleotide repeat, or simple sequence length polymorphism (SSLP), was discovered immediately proximal to the repeat, leading to the discovery that most patients had a contraction on one of the most prevalent 4A haplotypes with an SSLP length of 161 base pairs (4A161) [40]. Contractions on other 4A haplotypes like 4A159 and 4A168 were also associated with FSHD, with the notable exception that contractions on 4A166 chromosomes were not associated with FSHD [40,41]. Subsequent sequencing of the proximal and distal ends of the D4Z4 repeat array and its flanking sequences on these informative haplotypes allowed the identification of a series of SNPs and other polymorphisms that were consistently found on FSHD-permissive haplotypes, but not on non-permissive haplotypes [41].

The most striking difference between permissive and non-permissive haplotypes was the polymorphic nature of the *DUX4* poly-adenylation sequence previously identified [29,30]. While permissive haplotypes consistently contained a poly-adenylation sequence for the DUX4 mRNA, non-permissive haplotypes carried SNPs that disrupted this poly-adenylation sequence. This demonstrated that polymorphisms in the *DUX4* poly-adenylation sequence accounted for the restricted haplotype specificity of FSHD.

Finally, detailed genetic analysis of FSHD1 families with D4Z4 repeat array contractions that consisted of mixtures of 4-type and 10-type units showed that all pathogenic repeat arrays ended with a classical FSHD-permissive 4A sequence signature containing the *DUX4* poly-adenylation sequence, even when translocated to chromosome 10 [65]. Together, these studies showed that the DUX4 mRNA poly-adenylation sequence was necessary for FSHD, providing genetic proof that the DUX4 mRNA was necessary for FSHD.

### FSHD2: Genetic confirmation of decreased epigenetic repression as the cause of FSHD

The first evidence for an epigenetic disease mechanism in FSHD came from D4Z4 CpG methylation studies [66]. Making use of the diagnostic p13E-11 probe and a combination of methylation-insensitive and methylation-sensitive endonucleases, it was shown that the contracted repeat array was hypomethylated compared to normal-sized arrays [66,67]. However, FSHD2 individuals had a classical FSHD phenotype and did not have a D4Z4 repeat array contraction, but did show a strong reduction of D4Z4 methylation, suggesting that the common feature of FSHD1 and FSHD2 was decreased epigenetic repression of D4Z4 [66,68]. Methylation analysis of the D4Z4 repeat array in FSHD2 patients by bisulfite sequencing confirmed that the D4Z4 repeat is hypomethylated but that this hypomethylation is not uniformly observed across the entire unit [69].

Follow-up studies in a larger series of FSHD2 individuals showed very low D4Z4 methylation levels at the repeats of all four chromosomes but each affected individual had at least one FSHD-permissive chromosome 4 haplotype. Also other changes in epigenetic markers of the D4Z4 repeat, such as the binding of heterochromatin protein 1 gamma, the cohesin complex and some histone modifications, were similarly affected [70,71].

D4Z4 methylation analysis in family members of FSHD2 individuals showed that D4Z4 hypomethylation segregated as a dominant trait independent from disease presentation in some of these families. Digenic inheritance of the D4Z4 hypomethylation trait with an FSHD permissive allele was necessary for FSHD2, whereas individuals with hypomethylated non-permissive chromosomes 4 alleles remained unaffected [4].

Subsequent whole exome sequencing in selected FSHD2 families identified mutations in the *Structural Maintenance of Chromosomes Hinge Domain Containing 1* (*SMCHD1*) gene in six out of seven cases [4], and analysis of a larger cohort confirmed that *SMCHD1* mutations account for approximately 85% of FSHD2 families. In mice, Smchd1 was shown to be involved in gene repression by the establishment and/or maintenance of CpG methylation at loci that are monoallelically expressed, such as a restricted set of genes on the inactive X chromosome in females and the protocadherin gene cluster [72-75].

Analogous to the studies in mice, in muscle cell cultures from FSHD2 individuals and controls it was shown that SMCHD1 binds to the D4Z4 repeat and that there is reduced SMCHD1 binding in *SMCHD1* mutation carriers. Knock down and exon skip experiments confirmed that in control muscle cells containing an FSHD-permissive chromosome the reduction of SMCHD1 levels led to the derepression of *DUX4*, providing a mechanistic link between mutations in *SMCHD1* on chromosome 18 and *DUX4* reactivation from the D4Z4 repeat on chromosome 4 [4]. Thus, FSHD2 is caused by the digenic inheritance of a normal-sized D4Z4 repeat array on a *DUX4* poly-adenylation sequence containing chromosome 4 and a heterozygous *SMCHD1* mutation on chromosome 18.

Genetic variation in *SMCHD1* not only explains the majority of FSHD2 individuals, but contributes to the variability in onset and progression of the disease in some FSHD1 families. Examination of three FSHD1 families with a borderline D4Z4 repeat of 9 units and an unusually severely affected proband in each family, showed that the more severely affected proband had both the D4Z4 contraction causing FSHD1 and a mutation in *SMCHD1* causing FSHD2 [76], indicating that *SMCHD1* mutations modify the penetrance or severity of FSHD1 mutations. Indeed, knockdown of SMCHD1 in FSHD1 muscle cells led to an increase in DUX4 mRNA and its targets, indicating that both mutations act synergistically, and that FSHD1 and FSHD2 result from chromatin relaxation and derepression of *DUX4*. It will be interesting to explore to what extent natural genetic variation in the *SMCHD1* locus contributes to clinical variability in a larger cohort of FSHD1 families.

### A developmental model for FSHD

Increasing evidence indicated that FSHD was caused by the decreased epigenetic repression of the D4Z4 array and the variegated expression of the DUX4 mRNA in FSHD muscle, but neither the normal biological role of DUX4 nor the consequences of its expression in skeletal muscle were known. The retrotransposition of the *DUXC* gene created the *DUX4* retrogene at the root of the primate lineage [77]. Primates have maintained *DUX4* and lost *DUXC*, suggesting that DUX4 might have replaced the function of DUXC and might have also had a selective advantage for primates.

To become a retrogene, the parental gene must be expressed in the germline, and indeed *DUXC* is expressed in the canine testis (SJT, unpublished data) and the *DUX4* retrogene is expressed in the human testis [61]. Immunodetection showed DUX4 in luminal cells of the testis, consistent with spermatogonia and primary spermatocytes. Although additional studies are necessary to confirm the cell type expressing DUX4 in the testis, these results are consistent with expression of both DUXC and DUX4 in germline cells.

In contrast to skeletal muscle, DUX4 mRNA transcripts from both FSHD-permissive and non-permissive alleles are identified in testes. Testes DUX4 transcripts from both chromosome 4 and chromosome 10 splice exon 2 to novel distal exons and use a poly-adenylation sequence that is approximately six kb telomeric to the exon 3 poly-adenylation sequence used in FSHD muscle [61]. No skeletal muscle transcripts, either in control or FSHD tissues, have been identified that use these distal exons and poly-adenylation sequence and they appear to be testis specific, permitting stable DUX4 mRNA expression from both FSHD-permissive and non-permissive alleles in the testis.

Therefore, DUX4 appears to be expressed in the germline and epigenetically repressed in somatic tissues, likely through a repeat-mediated epigenetic silencing pathway. FSHD is caused by the inefficient epigenetic repression in skeletal muscle and the low-level variegated expression of the DUX4 mRNA and protein. This developmental model is supported by the demonstration that iPS cells from either FSHD or control individuals express the DUX4 mRNA, whereas DUX4 expression is suppressed in embryoid bodies derived from control cells and persists in embryoid bodies derived from FSHD cells [61].

### Consequences of DUX4 expression in skeletal muscle

DUX4 is a member of the double-homeobox family of transcription factors that arose in placental mammals. The role of DUX4 and this family of transcription factors in developmental biology remains unknown, but the consequences of the expression of DUX4 in skeletal muscle has been described. Earlier studies showed that expression of DUX4 in muscle cells induces apoptosis [78] that is dependent on an intact p53 [79]. An expression array and ChIP-seq analysis of human skeletal muscle cells transduced with DUX4 showed that DUX4 bound to a consensus sequence containing two homeodomain motifs and activated transcription of several hundred genes [80]. Consistent with a role in germline biology, DUX4 activated expression of a large number of genes normally expressed in the testis and in stem cell biology, as well as genes associated with RNA and protein processing, such as splicing factors and ubiquitin ligases.

Many expression array studies and some protein expression studies have been performed on control and FSHD muscle biopsies, but a strong molecular signature has been elusive. Some studies have focused on elevation of specific genes, such as *CRYM* or *PITX1*[30,81], or aspects of the program of muscle differentiation [82], or a molecular signature based on multiple genes without a clear regulatory association [83]. The recognition that DUX4 is expressed in a variegated pattern in cultured FSHD muscle cells might explain some of the difficulty identifying a clear gene expression signature when testing either cultures of FSHD cells or biopsies from FSHD muscle where DUX4 might be expressed in only a minority of the nuclei. Indeed, when expression array data are analyzed based on *a priori* knowledge of genes induced by DUX4-transduction in skeletal muscle cells, there is a significant elevation of these genes in FSHD muscle ([83] and SJT unpublished data); and a focused analysis shows that DUX4 and DUX4 target genes are expressed in fetal FSHD muscles [84].

**Figure 2:**
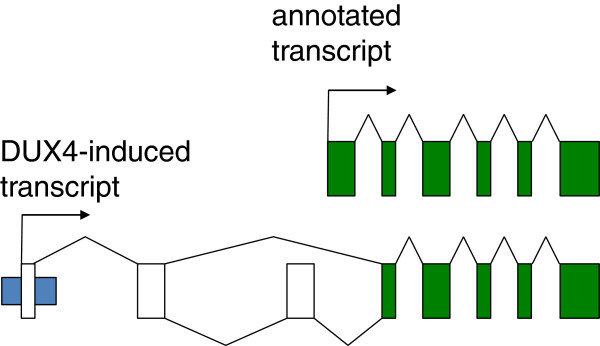
**Schematic of novel transcription start sites regulated by DUX4.** Upper panel shows the standard transcriptional start site for HEY1 and its intron-exon structure in green. The lower panel shows the transcript induced by DUX4 that originates in a retrotransposon LTR (blue) and splices into novel exons (uncolored) and the annotated HEY1 exons (green) to produce a predicted protein similar to HEY1.

Approximately one-third of DUX4 binding sites are in repetitive elements [85,86]. DUX4 binds the LTR element of the MaLR and HERVL family of retrotransposons and activates transcription of these endogenous repetitive elements. Interestingly, some of the genes that DUX4 regulates are initiated from the LTR elements of these retrotransposons (Figure 2). These retrotransposon subfamilies were last mobilized at the root of the primate lineage, at roughly the same time that *DUX4* retrotranposed from *DUXC*. These new primate-specific binding sites created a primate-specific gene network for DUX4, in addition to the network that evolved for DUXC prior to the last mobilization of these repeats in primates. It is important, therefore, to recognize that expression of DUX4 in non-primate cells will not recapitulate the gene network activated in human cells, consistent with the observation that there is only a partial overlap of genes regulated by DUX4 in human and mouse cells [86,87]. Therefore, murine models of the transcriptional activity or pathophysiology of human DUX4 expression need to be carefully designed and interpreted.

### Candidate mechanisms of disease

The consequences of DUX4 expression in skeletal muscle cells suggest several viable pathophysiological mechanisms for FSHD. Skeletal muscle cell apoptosis is perhaps the most dramatic consequence of DUX4 expression [78]. The apoptosis appears p53-dependent [79] but the specific molecular pathways remain to be defined. As noted above, DUX4 activates a stem-cell-like transcriptional program in skeletal muscle cells and it is possible that the incompatibility of these two differentiation programs is sufficient to induce an apoptotic response, similar to the older observations that promoting cell cycle in differentiated muscle cells leads to apoptosis [88]. Apoptotic loss of muscle nuclei that accumulates over time might therefore contribute to FSHD pathophysiology. In addition, DUX4 alters expression of genes involved in RNA splicing and processing, ubiquitin ligases implicated in atrophy, and many non-coding RNAs, any or all of which might contribute to FSHD pathophysiology.

In addition to damaging skeletal muscle, the expression of DUX4 might also inhibit normal muscle regeneration. DEFB103 is strongly activated by DUX4 in skeletal muscle cells [80]. DEFB103 is a member of the defensin family of small peptides and has been shown to inhibit the innate immune response and also to be an antagonist ligand for the CXCR4 receptor [89-91]. CXCR4 is expressed in activated muscle satellite cells and myoblasts. CXCR4 signaling has been shown to be necessary for myoblast migration and muscle cell differentiation [92,93], and exogenous DEFB103 inhibits muscle cell differentiation and the innate immune response in skeletal muscle cells [80]. Therefore, the induction of DEFB103 by DUX4 might prevent normal muscle regeneration and/or modulate the response of skeletal muscle to an immune stimulus.

A plausible model for a primary immune response as a component of FSHD pathophysiology also emerges from the identification of DUX4 regulated genes. Proteins expressed only in the germline can escape surveillance by the immune system since the germline is an immune-privileged site similar to the brain, and induce an immune response when mis-expressed in somatic tissues. This has been well studied in cancer biology where the mis-expression of germline genes induces an anti-cancer immune response. This group of genes is collectively referred to as cancer-testis-antigens. Therefore, the DUX4-induced mis-expression of these genes in FSHD muscle would be expected to induce an immune response. Whether this is the basis of the immune cell infiltrate associated with FSHD histology remains to be determined.

Interestingly, imaging studies in FSHD also support the possibility that an immune process might have a primary role in its pathophysiology. Recent studies have revealed an association between MRI characteristics consistent with an inflammatory process and the presence of infiltrating T-cells in FSHD muscle [20]. Then a subsequent study identified DUX4 regulated genes as mis-expressed in some FSHD muscles with inflammatory MRI characteristics [94]. Together with other similar studies that indicate edematous/inflammatory MRI characteristics progress to fatty infiltration [95-97], these studies suggest the possibility that DUX4 target gene expression and T-cell infiltrates might correlate with inflammatory characteristics on MRI. Further studies correlating the molecular characteristics of FSHD muscle with early MRI changes will be revealing in this regard.

Another broad category of DUX4-induced transcripts that might contribute to FSHD pathophysiology are repetitive elements, non-coding RNAs, and novel first exons originating in remnants of retrotransposon sequences [85]. DUX4 massively induces the pericentromeric satellite HSATII sequences that are also over-expressed in many cancers [98] and activates expression of endogenous retrotransposons and transcripts driven by isolated LTR elements that are the remnants of retrotransposons [85]. These RNAs and novel protein encoding transcripts could have biological activity related to FSHD pathophsysiology and need to be studied further.

An important observation was that some unaffected relatives of FSHD individuals express DUX4 mRNA in their skeletal muscle cells [99]. Although much more needs to be done to characterize these individuals, such as measuring the DUX4 protein and target genes, this observation suggests that other factors are necessary to initiate the disease process. Determining whether these are modifier loci that protect the muscle cell in a cell-autonomous or non-autonomous manner, for example, by preventing apoptosis or modulating an immune response, will greatly advance the understanding of FSHD pathophysiology.

### Therapeutic opportunities

The emerging consensus on mechanisms of FSHD pathophysiology greatly clarifies the approach to therapeutic interventions. The opportunities are clear: (1) enhance the epigenetic repression of the D4Z4; (2) target the DUX4 mRNA, including altering splicing or polyadenylation; (3) block the activity of the DUX4 protein; or (4) inhibit the DUX4-induced process, or processes, that leads to pathology.

Drugs that decrease epigenetic repression are now in wide clinical use, such decitabine, an inhibitor of DNA methylation, or SAHA, an inhibitor of histone de-acetylases; yet pharmaceutical companies have not prioritized the development of drugs that enhance epigenetic repression, partly due to the concern that these drugs would also suppress the class of tumor suppressor genes, such as p16, that are epigenetically repressed in many cancers. Although this was a reasonable concern, the demonstration that there are distinct molecular pathways for repeat-mediated epigenetic repression that do not target the more studied CpG island repression [71-74] suggests that this opportunity should be more actively explored. Furthermore, there is substantial support that the normal repeat-mediated repression pathways function through small RNA intermediates. The generation of small RNAs from the D4Z4 region [29] suggests that these might be mediators of the D4Z4 epigenetic repressive pathway and might be exploited to silence this region.

A more conventional approach would be to target the DUX4 mRNA and prevent it from making the DUX4 protein. Oligonucleotide-based therapies have already been developed for Duchenne muscular dystrophy [100], myotonic dystrophy [101], and other diseases [102,103], and many biotechnology companies are focusing on these approaches for a multiplicity of diseases. Their application to FSHD is both inevitable and welcome as a possibly rapid entry to clinical trials. In addition, some companies have shown the feasibility of screening for small molecules that might alter RNA splicing or poly-adenylation of a specific transcript, and this is also a possible approach for FSHD therapies.

While there are few drugs that block the activity of transcription factors, there are some examples [101] and drugs that block kinase cascades that might modify transcription factor activity are well known. Therefore it is not unreasonable to suppose that a drug blocking DUX4 transcriptional activity might be identified.

Currently, it is more difficult to imagine a roadmap to therapy by targeting a specific pathogenic pathway induced by DUX4, largely because there is no method to prioritize the multiple pathways that might lead to the FSHD pathophysiology. Each of these pathways might be effectively targeted by candidate therapies, but a human trial seems necessary to determine their relative importance. In contrast to the strong therapeutic justification for preventing the expression of DUX4 or its activity as a transcription factor, targeting individual pathways downstream of DUX4 will be partly an experiment to determine their relative role in disease pathogenesis.

### Cell and animal models

The earlier models for FSHD focused on the possibility that one of the closely linked genes might be deregulated in FSHD muscle. For some time the *Myd* mouse was considered a strong candidate because the *Myd* locus mapped to an apparently syntenic region on mouse chromosome 8 [104], but this was soon discarded as a model for FSHD with the identification of a mutation in the glycosyltransferase Large [105].

As mentioned, cellular models were first used to test the effects of DUX4 expression in a myogenic context. Transient transfection studies of D4Z4 in human cells showed that DUX4 is a nuclear protein that, when overexpressed, induces cell death [78]. An isogenic myoblast expression screen showed that DUX4 expression not only causes repression of glutathione redox pathway components and sensitivity to oxidative stress, but also repression of MyoD and MyoD targets with impaired myogenic differentiation. These effects could be neutralized by overexpression of Pax3 or Pax7 [106]. Detailed analysis of the transcriptional landscape of D4Z4 further identified an internal DUX4 methionine producing a C-terminal DUX4 polypeptide that is sufficient to inhibit myogenesis *in vivo* at a step between MyoD transcription and the activation of MyoD target genes [29].

Many other cellular models addressed the consequence of DUX4 expression, either by ectopically expressing DUX4, or by comparing FSHD muscle cell cultures with controls [84,86,107-110]. These cell models are also increasingly used for the identification of molecules that can suppress DUX4 activity [111,112], and to understand the regulation of DUX4 expression and activity [59,113-115].

Taking advantage of the high frequency of somatic mosaicism for the D4Z4 repeat array contraction, isogenic clonal muscle cell lines were established from a muscle biopsy of a mosaic FSHD individual that only differ by the presence or absence of a D4Z4 repeat array contraction [107]. These cell lines were useful to demonstrate that the burst-like feature of DUX4 expression is an intrinsic feature of the D4Z4 locus and, for the first time, showed that immortalized FSHD muscle cells can participate in muscle regeneration *in vivo* in immunodeficient host mice to overcome some of the limitations caused by the hominoid-specific features of DUX4. This was elegantly supported by a human skeletal muscle xenograft model in which human muscle biopsies were transplanted into muscle of immunodeficient host mice [116]. Muscle biopsies of FSHD individuals were shown to fully integrate and to express known human DUX4 biomarker genes.

Studies in zebrafish and *Xenopus* confirmed the toxicity of DUX4 *in vivo*[29,117] and its overexpression in muscle of zebrafish by transposon-mediated transgenesis or mouse muscle by adeno-associated viral vectors demonstrated the DNA binding-dependency of DUX4 toxicity [79,117,118]. This toxicity was also dependent on p53, as both *in vitro* and *in vivo*, DUX4 toxicity was overcome by the absence of p53. In an independent study, microinjection of small amounts of human full-length DUX4 mRNA into fertilized zebrafish eggs caused features reminiscent of FSHD such as abnormalities of fin, facial, and trunk muscles, and mislocalization of myogenic cells outside somite boundary. Interestingly, these abnormalities could be rescued by introducing DUX4-s that lacks the putative DUX4 transactivation domain [118]. Moreover, DUX4-induced damage in the mouse muscle seems to be reversible as RNA interference (RNAi)-based DUX4 therapy corrected the myopathic changes caused by DUX4 expression in the muscle [119].

In a more recent study, a different approach was taken by introducing the entire FSHD1 locus in the mouse genome [87]. One mouse line had a D4Z4 repeat array of 2.5 units (D4Z4-2.5), while the second line carried an array of 12.5 units together with centromeric DNA that included the *FRG2*, *DUX4C*, and *FRG1* loci. While neither of these mice produced a clear muscle phenotype, these mice do recapitulate many of the D4Z4 regulatory mechanisms first reported in humans. D4Z4-12.5 mice have a repressed D4Z4 chromatin structure in somatic cells and only express DUX4 at consistently detectable levels in germline tissues while in D4Z4-2.5 mice, there is a partial opening of the chromatin structure similar to FSHD patients and derepression of DUX4 leading to the molecular hall mark of the disease with few nuclei expressing high amounts of DUX4 protein. A possible explanation for the lack of muscle pathology could be that human DUX4 does not activate the same gene network in murine cells, in part due to the retrotransposon-mediated spread of its binding sites in primates as discussed above, and possibly for other reasons yet to be determined.

### FSHD: A consensus model with minor areas of disagreement

There is increasing consensus on the primary disease mechanism of FSHD1 and FSHD2. Since the publication of the unifying genetic mechanism predicting a prominent role for the distal copy of the *DUX4* retrogene and the polymorphic *DUX4* polyadenylation sequence [65], efforts are largely focusing on the D4Z4 chromatin changes and the consequences of somatic DUX4 expression [60,69,71,80]. Also, the identification of the FSHD2 gene, *SMCHD1*[4,76], causing similar changes in D4Z4 chromatin structure, and derepression of *DUX4*, strongly support the involvement of polyadenylated DUX4 transcripts in FSHD pathophysiology.

However, there are still observations and areas of disagreement that need to be addressed. Based on the SSLP immediately proximal to the repeat, a number of chromosome 4A and 4B subclasses were identified, of which a subgroup was shown to be permissive for FSHD (most commonly 4A161). All permissive subclasses have the polymorphic *DUX4* polyadenylation sequence. One notable exception is the 4A166 haplotype. In a Dutch study [56], D4Z4 contractions on the 4A166 haplotype did not result in FSHD despite the presence of the *DUX4* polyadenylation sequence. An Italian study [11], however, identified a considerable number of individuals with FSHD having a contraction on the 4A166 haplotype, concluding that short D4Z4 repeats on this haplotype can be pathogenic. However, the frequency of the 4A166 haplotype in the population was very different in both studies suggesting that perhaps a technical issue with the determination of the haplotype may be the cause of this apparent discrepancy. Despite this controversy about the pathogenicity of the 4A166 haplotype, both studies showed that the great majority of FSHD1 patients have a D4Z4 contraction on an FSHD-permissive allele defined by the presence of the *DUX4* polyadenylation sequence. One direction of future research might be to investigate the role of disease haplotype-specific sequence variants in addition to the DUX4 polyadenylation sequence in the processing of DUX4 mRNA to better understand the essential genetic features of an FSHD permissive allele.

Another area of future research is the role of proximally located genes such as *FRG1*, *FAT1*, and *DUX4C* in FSHD pathogenesis. Although the genetic data in FSHD families do not provide strong support for a critical role for these genes and point to a primary role for the distal unit of the D4Z4 repeat, some studies have provided evidence for deregulation of proximally located genes in FSHD [51,52,120,121]. In addition, studies in cell and animal models provided evidence that overexpression of these proteins can lead to muscle cell pathology. One of the best studied mouse models is a transgenic animal overexpressing human *FRG1* at different levels in skeletal muscle [122]. This mouse developed an FRG1 dose-dependent muscle pathology. Another intriguing mouse model with a deregulation of the expression of the protocadherin gene *FAT1* (located several megabases centromeric to D4Z4) also shows a muscle pathology [123]. Also in FSHD fetuses, *FAT1* expression seems to be disturbed but whether this is a direct consequence of D4Z4 contraction [119], or perhaps a more downstream effect remains to be established. The absence of consistent data that these genes are indeed deregulated in FSHD muscle challenges their involvement, although the FRG1 mouse model was useful for a proof-of-concept study that knocking down of FRG1 in these mice by viral shRNA delivery prevented or restored muscle pathology [124].

The role of non-coding RNAs in the regulation of the D4Z4 chromatin structure also needs further studies. While there is agreement that the D4Z4 region is broadly transcribed and produces both sense and anti-sense RNAs [29], one study found these transcripts from D4Z4 arrays on chromosomes 4 and 10 and from both wild-type and disease-associated alleles and suggested that these RNAs and the small RNA fragments generated from them might be involved in epigenetic silencing of the region [29]; whereas another study found a long-RNA transcript specifically from the contracted allele and showed that it had a derepressive role in the region [60]. Further studies will be necessary to reconcile these apparently different conclusions.

Finally, variability in onset and progression is one of the clinical hall marks of FSHD, already noted in the first description of FSHD, and recently further highlighted in a large genotype-phenotype study [125]. Some attempts have been made to correlate the phenotype with the residual D4Z4 repeat size and with epigenetic changes of the repeat [71,126-128]. These studies show that patients with 1 to 3 units are typically severely affected cases while the disease spectrum in individuals with 4 to 10 units can vary from asymptomatic gene carriers to severely affected patients. The cause for this wide variability in clinical presentation is still poorly understood. A recent paper by Sacconi *et al.*[76], showing that mutations in the FSHD2 gene *SMCHD1* act as a disease modifier in FSHD1 families suggests that epigenetic variation may, at least in part, underlie the clinical variability of FSHD. It will be important to further study the role of SMCHD1 and other epigenetic modifiers in relation to clinical variability of FSHD.

## Conclusions

Although some areas of disagreement remain, there is increasing agreement and consensus on a model of FSHD pathophysiology that accounts for nearly all of the current experimental findings. This model is based on the inefficient epigenetic repression of the *DUX4* retrogene in the D4Z4 macrosattelite repeat adjacent to a polymorphic poly-adenylation sequence in the subtelomeric region of chromosome 4, and results in variegated expression of DUX4 in skeletal muscle nuclei in FSHD. The genes regulated by the DUX4 transcription factor suggest several plausible mechanisms of disease pathophysiology, including apoptosis, inhibition of regeneration, and a primary immune response. The direct transcriptional consequences of the expression of the DUX4 transcription factor in postmitotic muscle are becoming known, and current efforts are focusing on translating this knowledge towards identification of useful biomarkers and understanding the pathophysiological consequences. Further pre-clinical and clinical studies will be necessary to determine whether FSHD results from a single dominant pathway modulated by DUX4, or whether it reflects damage from multiple different pathways. Whichever is the case, the development of a generally accepted and coherent model for FSHD provides new avenues for therapeutic development. Although no single FSHD animal model exists besides humans, multiple different cell and non-human animal models that embody distinct components of the pathophysiological pathways have already been developed and can be used for testing candidate therapies. In summary, we have reached a pivotal milestone in FSHD research when the field is coming to agreement on a consensus model of pathophysiology and can confidently use this model as the basis for therapeutic development. Based on the rapid advances in FSHD research and the new efforts at drug development, it is safe to conclude that clinical trials are just around the corner. A major challenge for the coming years will be to put in place the elements needed for clinical trials, which will include: (1) establishment and maintenance of patient registries based on globally accepted datasets; (2) development and validation of patient-relevant outcome measures; and (3) further development and validation of biomarkers, both molecular and radiologic [129].

## Abbreviations

FSHD: Facioscapulohumeral dystrophy; iPS: Induced pleuripotent stem cells; ORF: Open reading frame; RT-PCR: Reverse transcriptase polymerase chain reaction.

## Competing interests

The author(s) declare that they have no competing interests.

## Authors’ contributions

RT: Conception, manuscript writing, and final approval of the manuscript. SJT: Conception, manuscript writing, and final approval of the manuscript. SMvdM: Conception, manuscript writing, and final approval of the manuscript. All authors read and approved the final manuscript.
